# Component Analysis of Gas Mixture Based on One-Dimensional Convolutional Neural Network

**DOI:** 10.3390/s21020347

**Published:** 2021-01-06

**Authors:** Canjian Zhan, Jiafeng He, Mingjin Pan, Dehan Luo

**Affiliations:** School of Information Engineering, Guangdong University of Technology, Guangzhou 510006, China; zcj@mail2.gdut.edu.cn (C.Z.); illusionnol66@gmail.com (M.P.); dehanluo@gdut.edu.cn (D.L.)

**Keywords:** electronic nose, convolutional neural network, component analysis

## Abstract

Indoor harmful gases are a considerable threat to the health of residents. In order to improve the accuracy of indoor harmful gas component identification, we propose an indoor toxic gas component analysis method that is based on the combination of bionic olfactory and convolutional neural network. This method uses the convolutional neural network’s ability to extract nonlinear features and identify each component of bionic oflactory respense signal. A comparison with the results of other methods verifies the improvement of recognition rate while with the same level of time cost, which proved the effectiveness of the proposed model. The experimental results showed that the recognition rate of different types and concentrations of harmful gas components reached 90.96% and it solved the problem of mutual interference between gases.

## 1. Introduction

Because low-concentration indoor harmful gases are invisible and tasteless, they are difficult for people to distinguish. We can detect low-concentration indoor toxic gases through physical and chemical identification methods. Still, they are cumbersome and complicated operations, and to use the instrument needs to be professionally trained. It is difficult to promote in the market.

Many different methods are applied in indoor air environment monitoring for the quantitative analysis of harmful gases, including the non-dispersive infrared method [1], gas chromatography [2], nessler’s reagent colorimetry [3], and ion-selective electrode method [4]. The methods, as mentioned above, are relatively complicated and they cannot perform real-time on-site air quality testing. With the rapid development of information science and sensor technology, the bionic olfactory system has been applied in medical, food processing, and environmental detection fields, with its advantages of simplicity and economy.

However, when machine olfactory technology is used in the quantitative analysis of substance odor in an open environment, it is easily affected by interfering gases and environmental temperature and humidity, which causes the problem of reduced recognition accuracy.

The machine learning algorithms and their optimization methods were applied to the quantitative analysis of machine olfactory rapidly. For example, Xianjiang Li et al. proposed an optimization model for mixed gas quantitative detection that combines an adaptive genetic algorithm and a traditional BP neural network. This algorithm can overcome the shortcomings of the slow search rate and easily fall into local minimum. The BP neural network can obtain better initial weights and early stage thresholds through the adaptive genetic algorithm. Subsequently, the experiment uses this algorithm to quantitatively analyze the machine olfactory odor data of a set of five-element gas mixtures. The experiment shows that the accuracy of the algorithm for identifying the gas concentration of the gas mixture is higher than that of the traditional BP neural network [5]. Shurui Fan et al. used principal component analysis and random forest modeling, which can qualitatively identify methane and ethylene gases. Subsequently, the optimal regression model is constructed through support vector regression and particle swarm optimization for quantitative analysis of the two types of gases. The experimental results show that the average recognition rate of principal component analysis, combined with random forest, is 97% higher than logistic regression and the support vector machine. The fitting effect of support vector regression is optimized by the particle swarm algorithm, and better fitting results are obtained than support vector regression [6].

In practical applications, when using machine olfaction to quantitatively analyze the odor of substances, it is often accompanied by the influence of interfering gas. Therefore, nonlinear or linear inseparable phenomenon, or even gas shielding, will often appear in the machine olfactory system. The phenomenon of shielding between gases means that the response of the electronic nose sensor when measuring mixed gas is lower than that of measuring pure gas of the same concentration [7]. However, the above-mentioned literature does not fully consider the influence of these phenomena on the quantitative analysis of machine olfaction [8], so some improved algorithms are proposed.

Yu Lu et al. combined the artificial neural network with the basic concepts of analytical chemistry, designed an error function based on analytical chemistry, and applied the error function to the neural network. This method can be used to control alcohol, petroleum gas, and water. The experimental results show that the method predicts a gas concentration error less than 10% [9]. Tang K TZ et al. proposed a Locally Weighted Nearest Neighbor (LWNN) algorithm that is based on the K-Nearest Neighbor classifier (KNN) algorithm to determine the odor components, and then combined with the types of odor components, while using the weighted and constrained least squares (Weighted and Constrained Least-Squares, WCLS) gas concentration estimation method that is based on the least squares method measures the target gas concentration in the mixed gas [10].

The above analysis shows that, in addition to investigate the feasibility of applying machine odor perception to quantitative gas analysis, great progress has been made in the component analysis methods of machine odor perception; however, there are still various shortcomings. The methods are not universally applicable and they do not take the mutual influence between different gas concentrations and the reversal of the response curves into consideration. Therefore, none of the above methods can be used for the component analysis of indoor pollutant gases while using the odor perception engine. It is necessary to find appropriate methods that are based on the properties of the pollutant gas odor data for indoor spaces in order to extract the component properties of the target gas (for example, formaldehyde) among other indoor gases. Based on the background values of indoor air pollution, this paper proposes a component analysis method for indoor pollutant gases when considering the influence of interfering gases.

## 2. Gas Data Preprocessing

According to the requirement of the research objectives and content of this article, a machine olfactory system is required for collecting the odor information of indoor harmful gases. This article contains the odor data of multiple indoor toxic gas samples through the PEN3 electronic nose system in order to ensure the reliability of the data. The PEN3 electronic nose is the third generation of the PEN series developed by AIRSENSE, Germany. It is built with 10 cross-sensitive metal oxide gas sensors. Table 1 shows the characteristics of the sensors in PEN3 electronic nose.

**Figure 1:**
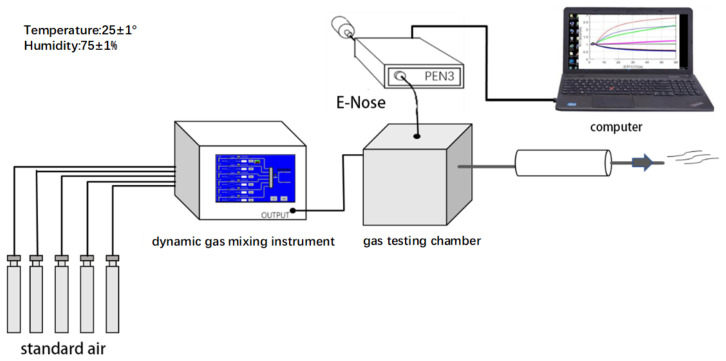
The experimental procedure of indoor hazardous gas collection based on machine olfaction.

This article selected the four most common pollution gases in daily life based on the needs of the research objectives and content of this article. They are formaldehyde, ammonia, benzene, and methanol, produced by Henan Testing Center. Additionally, the multi-channel gas mixing system performs the ratio of indoor harmful gases in order to ensure the objectivity and reliability of the data. Appropriate experimental materials and exhaust gas treatment equipment should be selected when designing the practical plan in order to ensure the safety of the experiment. Figure 1 shows the process of harmful indoor gas collection based on machine olfactory. In order to obtain accurate gas concentration sample data, the MT-500X dynamic gas mixing system (gas mixing instrument) is used in this paper to carry out the precise ratio of indoor harmful gas samples, to ensure the objectivity and accuracy of the data that were collected in this paper. The system is equipped with a high-precision mass flow controller, which can meet the requirements of stable, reliable, and high-precision gas distribution.

Before starting the experiment, the indoor air conditioner and humidifier should be turned on, so that the temperature should be controlled within the range of 25 ± 1 °C and the humidity should be controlled at 75 ± 1%. Table 2 shows the other experimental parameters. First, we prepare the gas samples that are required for the experiment with the standard gas through the gas mixing instrument, transport the prepared gas samples to the gas testing chamber, and then collect the odor data through the PEN3 electronic nose. Finally, the residual test gas in the gas test box is passed through the tail gas treatment device for harmless treatment.

**Figure 2:**
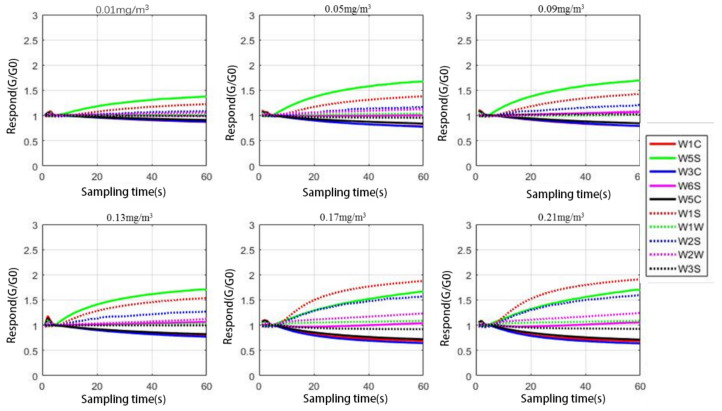
The response curve of weak interference group.

**Figure 3:**
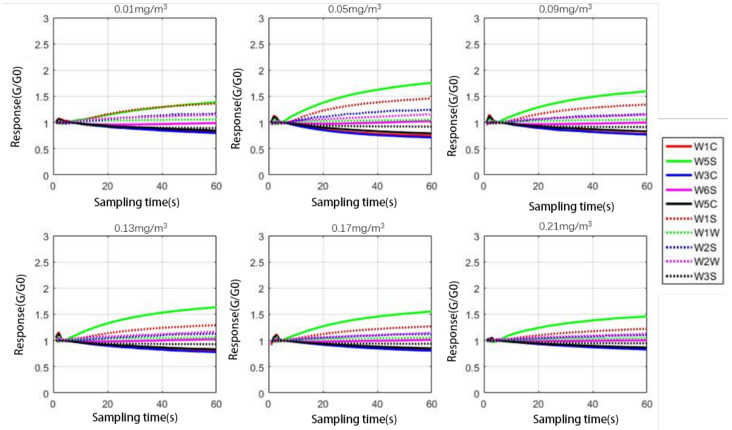
The response curve of moderate interference group.

According to different interference groups, the response curve of the electronic nose to different indoor harmful gases in the same interference group can be drawn through the data set. The concentrations of formaldehyde gas containing 0.01 mg/m^3^, 0.05 mg/m^3^, 0.09 mg/m^3^, 0.13 mg/m^3^, 0.17 mg/m^3^, and 0.21 mg/m^3^ are drawn, respectively, as shown in Figure 2 and Figure 3.

**Figure 4:**
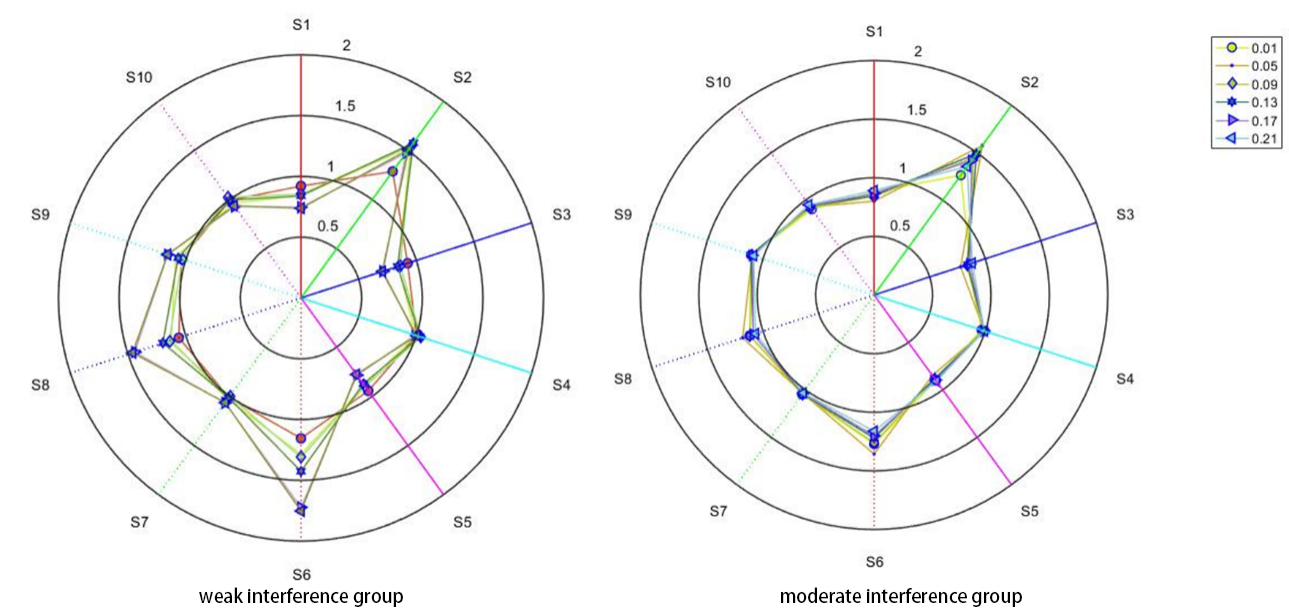
Radar diagram for each set of data.

By observing Figure 2 and Figure 3, it can be found that, in the case of the same interference group, each response curve of the sensor is very similar in the overall listing, and it is still necessary to use the radar chart to supplement the observation. The radar chart data still uses the data with a sampling interval of 30 s to 40 s in order to calculate the average value, and then the average value is converted into a radar chart, as shown in Figure 4.

The response values of sensors numbered S2, S6, and S8 are quite different from other sensors, especially for S2. The response of the sample is more sensitive. However, with the exception of the S2, S6, and S8 sensors, the difference in the response values of other sensors is small enough to determine that “high dimensionality, redundant information, and non-linearity” are the characteristics of indoor harmful gas data that are collected by machine olfactory.

The above data show that we can infer that indoor harmful gas samples containing different formaldehyde gas have differences in data that are based on the difference between the response curve of the sensor and the radar chart. According to their differences, suitable identification methods can be selected in order to train the computer to identify the level of formaldehyde pollution in indoor harmful gases.

In this experiment, Table 3 shows the 60 × 10 odor data matrix generated by PEN3. Each column represents a different sensor, and each row represents the response of the same sensor at different sampling times.

According to the original data format of the PEN3 electronic nose in Table 3, the data matrix of 60 × 10 is first transposed to the data matrix of 10 × 60, and then the data matrix is represented by a brand new row vector. The specific method is as follows. Based on the corresponding sensor that generates the response, the original matrix is divided into 10 row vectors of 60, and is then sequentially connected by the sensor number to form a new row vector, and stored in the .csv format. If the sampling time of the acquisition experiment is 60 s to collect m samples, then the converted data set of the electronic nose data file is a .csv file with m 600-dimensional features. In this paper, 1040 gas samples are collected through experiments, so the data in this paper are concentrated.

In this paper, a total of 1040 data samples were collected through the collection experiment. According to the indoor air quality standard (GB/T18883-2002), the data samples are divided into three types of data with different pollution levels. Among them, 320 data samples containing formaldehyde gas from the concentration of 0.01 mg/m^3^ to 0.08 mg/m^3^ (without 0.08 mg/m^3^) are qualified (Normal), and 320 data samples with the formaldehyde concentration from 0.08 mg/m^3^ to 0.16 mg/m^3^ were classified as mildly polluted (Mild), and 400 data samples containing formaldehyde concentrations that were greater than 0.16 mg/m^3^ were classified as severely polluted (Serious). Select 70% of the data samples of each type of pollution level as the training set and the remaining 30% data samples as the test set of the experiment. The data set used for model training has 728 data samples, and the data set used for testing has 312 data samples.

Therefore, we construct an indoor hazardous gas odor information collection system. A number of indoor harmful gas samples with different concentrations were prepared by a dynamic mixing gas distributor, and the PEN3 electronic noses was used in order to collect odor data from these samples, thereby obtaining a data set of the response of the indoor harmful gases. The exhaust gas of the experiment was treated in a harmless manner in order to eliminate the impact of harmful gases on the health of the experimenters. According to the characteristics of the electronic nose response data, a component analysis method of indoor harmful gases based on machine olfaction and global average pooling convolutional neural network model is proposed.

## 3. One-Dimensional GAP-CNN

CNN is a neural network with deep structure and a classic algorithm that is widely used in deep learning [11]. Nowadays, many typical and widely used CNN models have been proposed, such as LeNet-5 [12], AlexNet [13], and GoogleLeNet [14]. They have been successfully appled in face detection [15], role recognition [16], pedestrian detection [17], and robot navigation area [18]. Because CNN has the structural characteristics of local connection, weight sharing, and down-sampling, the model is sample-invariant to translation, scaling, and distortion, and, thus, has strong robustness [19]. This feature makes convolutional neural networks a great success in the field of image processing. The main difference between CNN and the traditional BP (Back Propagation, BP) neural network lies in the two aspects of weight sharing and local connection. Weight sharing makes the convolutional neural network more suitable for the structure of biological neural networks. The local connection of convolutional neural network is not like a traditional neural network. Each neuron in the first layer is connected to all neurons in the first layer, but the neurons in the first layer are partially connected to the neurons in the first layer. The role of these two characteristics makes the model have lower model complexity and fewer weights than traditional BP neural networks.

The convolution layer performs convolution processing on the input data through multiple convolution kernels and extracts the convolutional features, which is the feature map. A corresponding type of feature is extracted through a convolution kernel. Because the operation of the same convolution kernel has the characteristics of local connection, parameter sharing, and multiple convolution kernels, when compared with the fully connected layer, the convolution layer can propose more features with fewer parameters when extracting data features. Because the convolution structure is not affected by the input dimensions and the training depth structure is simple, it can effectively extract features from complex and high-latitude inputs. The convolution formula of the convolution layer is:

(1) $$g\left(i\right)=\sum_{x=1}^{m}\sum_{y=1}^{n}\sum_{z=1}^{p}\alpha_{x,y,z}\times w_{x,y,z}^{i}+\beta^{i},i=1,2,\ldots ,q$$

where: *i* is the *i*-th convolution kernel, *g*(*i*) is the feature map that is extracted by the *i*-th convolution kernel; α is the input data; β is the bias of the convolution kernel; *x*, *y*, *z* represent three different dimensions of data. After completing the convolution of the data, it is necessary to use a nonlinear activation function in order to perform nonlinear conversion on the data. The commonly used activation function in CNN is generally ReLU, and its formula is:

(2) y(i)=f(g(i))=max{0,g(i)},i=1,2,…,q

(3) $$p^{l(i,j)}=\max_{(j-1)w<t<jw}\left\{\alpha^{l(i,t)}\right\},j=1,2,\ldots ,q$$

(4) $$p^{l(i,j)}=\underset{(j-1)w<t<jw}{\mathrm{avg}}\left\{\alpha^{l(i,t)}\right\},j=1,2,\ldots ,q$$

where: $\alpha^{l(i,t)}$ is the *t*-th neuron of the *i*-th feature map in the *l*-th layer, *w* is the width of the convolution kernel, and *j* is the *j*-th pooling kernel [20].

In the convolutional neural network, the convolutional layer and the pooling layer both perform feature extraction on the data, and the operation of data classification is performed in the fully connected layer based on the feature. The fully connected layer can integrate features through the feature maps output by the convolutional layer, thereby obtaining classification information with high-level meaning, and then classify and output according to these. As the output of the CNN model, the output of the fully connected layer is a fixed-length feature vector that is obtained by transforming the feature map input from the layer. The feature information of all combinations of the original data will be integrated by this feature vector. Although this fixed-length feature vector does not have the location information of the original data, the feature information used to effectively complete the data classification task has been fully extracted [21].

In the traditional convolutional neural network, the parameter ratio of the fully connected layer is almost 80% of the entire neural network model [22]. In order to achieve the purpose of reducing network parameters, there are generally two common methods. The first method is to replace the fully connected layer in the model with a convolutional layer. If the convolution kernel of the same size as the feature map in the full connection layer is used as the input of the output layer, this often makes the model output result far inferior to using the fully connected layer as the input of the output layer accurate. The second approach is to reduce the feature output dimensions of all the layers in the network, but the disadvantage of this approach is that the neural network lacks sufficient features for network training, which results in the final output feature information that contains less feature information than the original dimension, which reduces the model accuracy of recognition.

In this article, we refer to the idea of using global pooling in GoogleNet, and use the global pooling layer to replace the fully connected layer in order to extract features [23]. There are generally two methods for global pooling, global average pooling (GAP) and global maxing pooling (GMP). This article takes the global average pooling as an example to illustrate the difference between global pooling layer and full connection. The global average pooling layer, like the fully connected layer, has the function of connecting global feature information, so as to ensure the accuracy of the output result of the recognition model. However, because the global pooling layer does not contain any parameters, the phenomenon where the fully connected layer is easy to overfit is avoided during the model training process.

## 4. Result

**Figure 5:**
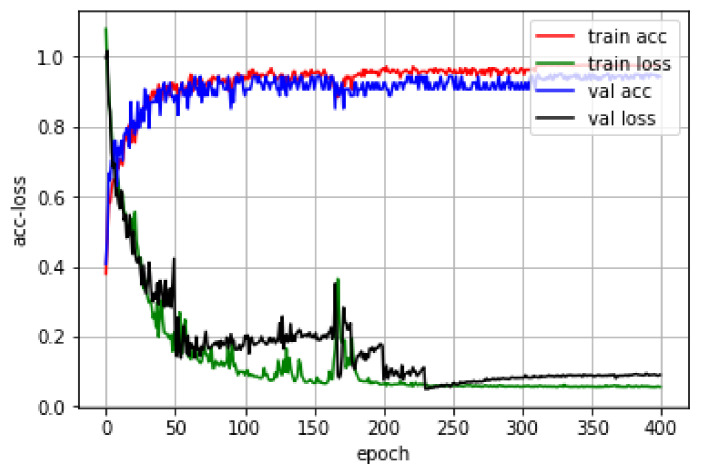
Training curve of global average pooling-CNN (GAP-CNN) model.

**Figure 6:**
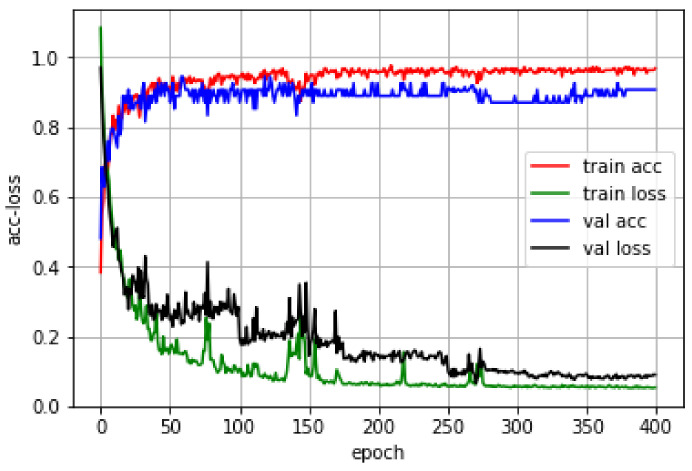
Training curve of CNN model.

In order to verify that GAP-CNN can reduce the weight parameters while ensuring the accuracy of identifying formaldehyde pollution in indoor harmful gases, a CNN model with the same depth and the same convolution kernel size as GAP-CNN will be used as the control group. Both network models use the same convolution layer and convolution kernel with the same parameter settings. However, in the CNN model, the number of parameters that need to be trained is 14,963, which is much higher than the number of 4259 parameters shown in the GAP-CNN model. The reason is that in the CNN model, the number of parameters in the first dense layer, which is the fully connected layer, accounts for 70% of the total network model parameters. This also proves that global pooling can effectively compress the volume of the convolutional neural network model. The parameters of the prediction models of the two convolutional neural networks are set as: the number of training data is epoch = 400, the size of the training set data and the test set data is randomly selected each time batchsize = 32, and the learning rate α is 0.01. Figure 5 and Figure 6 show the training curves of the two models, respectively.

Among them, the abscissa represents the number of epochs of model training, the ordinate represents the accuracy and the size of the loss function value, train acc represents the recognition rate of the model to the training set, train loss represents the value of the loss function of the model to the training set, the val acc model represents the recognition rate of the set, and val loss represents the value of the loss function of the model to the training set. In Figure 5, the values of train loss and train acc of GAP-CNN reach a stable state after the number of training is 260. The GAP-CNN model has been trained. The value of train acc of the model is 0.9684, and the value of train loss is 0.0954. The correct recognition rate val acc of the model on the training set is 0.9140. From Figure 6, the train loss and train acc values of the CNN model reach a stable state after 230 training times. The train acc value of the CNN model is 0.9615 and the train loss value is 0.1083. The correct recognition rate val acc of the CNN model on the training set is 0.9071.

When comparing the training curves of the two models, we can find that, under the same training parameters, although the convergence speed of the GAP-CNN network model is slightly slower than that of the classic CNN, the recognition of GAP-CNN on the test set of indoor harmful gases. The accuracy is slightly better than the classic CNN model, which also proves that the GAP-CNN network model can reduce the model parameters while reducing the occurrence of overfitting, and it can also ensure a certain model accuracy. By saving the GAP-CNN model to identify the test set again, it is found that there are three main reasons why the classification accuracy rate cannot reach 100%. The first is that GAP-CNN will make a small part of the formaldehyde concentration in the strong interference group close to 0.08 mg/m. The Normal class is incorrectly identified as the Mild class. The second reason is that GAP-CNN will incorrectly classify a small part of the Mild and Serious, especially gas samples whose concentration is at the classification boundary. For example, gas samples containing Mild’s 0.14 mg/m^3^ formaldehyde will be classified as Serious. The Serious class’ 0.18 mg/m^3^ will be classified into the Mild class. The third reason is that there is a high concentration of interfering gas, indoor harmful gas samples containing low concentration of formaldehyde gas, the model can easily identify it as a higher level of formaldehyde pollution level.

In order to further verify the effectiveness of the GAP-CNN algorithm, in Table 4, PLS model, PCA+LDA model, CNN model, t-SNE+RF model, and GAP-CNN model are used in order to calculate the average of the classification results while using five-fold cross-validation.

Through the analysis and research of the above experimental results, it can be known that the traditional PCA+LDA machine olfactory recognition method cannot effectively extract the effective features for the classification of formaldehyde pollution in indoor harmful gases. The PLS model can extract the effective features of formaldehyde pollution classification in indoor harmful gas to a certain extent, but, in the presence of other indoor harmful gas interference, the accuracy of algorithm recognition is low. The t-SNE+RF algorithm is less accurate than the classification that is based on the CNN neural network. In the CNN and GAP-CNN models, although the classification accuracy of the Normal level of formaldehyde pollution is not as good as t-SNE+RF, as compared with the t-SNE+RF algorithm, it is found that the advantage of the CNN neural network formaldehyde pollution classification is that CNN When the neural network recognizes the level of formaldehyde pollution of Mild and Serious, it has higher classification accuracy. At the same time, GAP-CNN has better generalization performance than t-SNE+RF.

By observing the results of cross-validation experiments, it can be found that CNN, t-SNE+RF, and GAP-CNN have an ideal effect on identifying the level of formaldehyde pollution in indoor harmful gases. Therefore, this paper uses the aboved algorithm to compare and analyze the total computing time of data preprocessing, feature extraction, model training, and output results in order to further compare the characteristics and performance of the three machine learning algorithms to identify machine olfactory odor data. The operation time presented in Table 5 shows that GAP-CNN algorithm needs to go through multi-layer convolution operation, and the convergence speed is slow, which leads to greater time consumption of GAP-CNN algorithm.

## 5. Discussion

This paper proposes an indoor harmful gas component analysis algorithm that is based on the combination of CNN and bionic olfactory. This method uses one dimensional convolutional neural network weight sharing and adding a global maximum pooling layer, so that the neural network has a higher recognition rate for indoor harmful gases when the number of training parameters is small. The research of this algorithm is of great significance to the solution of the subsequent concentration estimation problem of the bionic olfactory system. The algorithm that is proposed in this paper has not been well verified in the concentration regression experiment. In the process of the experiment, the influence of external factors on the experimental results has not been considered. This will be the direction of follow-up research.

## 6. Conclusions

In this work, a novel component analysis strategy was proposed for formaldehyde pollution in harmful indoor gases based on machine olfaction. While using a portable electronic nose system, indoor harmful gas samples’ odor information is collected and processed to achieve an real-time monitoring. By converting the smell information of the machine olfaction into a one-dimensional time series, the machine olfactory analysis method GAP-CNN that is based on global average pooling and one-dimensional convolutional neural network is innovatively proposed. Additionally, through the comparison of the experimental results, in the case of compressing the model volume, the recognition accuracy of the model is guaranteed, which proves the performance of the GAP-CNN model. At the same time, this paper uses the GAP-CNN model and the classic CNN model to build an indoor hazardous gas formaldehyde pollution classification model. Through comparing the experimental results, it is found that the accuracy of the two classification models is greater than 90%, and the weight parameters of the GAP-CNN model are much lower.

## Figures and Tables

**Table 1:** The characteristics of the sensors in PEN3 electronic nose.

The Number of Sensors	The Name of Sensors	Sensitive Substances (Primary Response Components)
S1	W1C	aromatic substances such as toluene
S2	W5S	nitrogen dioxide and other nitrogen oxides
S3	W3C	ammonia and aromatic substances
S4	W6S	hydrogen
S5	W5C	alkanes and aromatic substances
S6	W1S	methane
S7	W1W	sulfide
S8	W2S	ethanol
S9	W2W	aromatic substances and organic sulfur substances
S10	W3S	alkanes

**Table 2:** The parameters of the gas data acquisition experiment.

Experimental Parameters	Parameter Setting
collect temperature	25 ± 1 °C
collect humidity	75 ± 1%
gas sample volume	4 L
gas testing chamber volume	4 L
sample standing time	30 min
sensor array automatic cleaning time	120 s
sampling time	60 s
sample interval	1 s
gas-flow rate	60 mL/min

**Table 3:** Raw data format.

sensor1	sensor2	......	sensor10
1.0040	0.9695	......	1.0023
1.0224	1.0341	......	1.0065
.......	......	......	......
1.3567	1.1501	......	1.2354

**Table 4:** Cross validation results of 5 models.

Algorithm	PLS	PCA+LDA	CNN	t-SNE+RF	GAP-CNN
Accuracy (%)	71.16	41.54	90.58	85.58	90.96

**Table 5:** Computational time comparison of discrimination algorithm.

Algorithm	Computing Time (s)
CNN	19.3968
t-SNE+RF	12.1842
GAP-CNN	25.7133

## Data Availability

All data generated or appeared in this study are available upon request by contact with the corresponding author. Furthermore, the models and code used during the study cannot be shared at this time as the data also forms part of an ongoing study.

## Abbreviations

The following abbreviations are used in this manuscript:

CNN	Convolutional Neural Network
KNN	K-Nearest Neighbor
WCLS	Weighted and Constrained Least-Squares
KNN	K-Nearest Neighbor
GMP	Global Maxing Pooling
PCA	Principal Component Analysis
LDA	Linear Discriminant Analysis
GAP	Global Average Pooling
